# Cycloartane Glycosides from the Roots of *Cimicifuga foetida* with Wnt Signaling Pathway Inhibitory Activity

**DOI:** 10.1007/s13659-015-0053-7

**Published:** 2015-02-19

**Authors:** Di-Fan Zhu, Guo-Lei Zhu, Ling-Mei Kong, Ni-Man Bao, Lin Zhou, Yin Nian, Ming-Hua Qiu

**Affiliations:** State Key Laboratory of Phytochemistry and Plant Resources in West China, Kunming Institute of Botany, Chinese Academy of Sciences, Kunming, 650201 China

**Keywords:** *Cimicifuga foetida*, 9,19-Cycloartane triterpenoids, Cimilactone-type, Wnt signal pathway, Luciferase activity

## Abstract

Four new 9,19-cycloartane triterpenoids, cimilactone E (**1**), cimilactone F (**2**), 2′-*O*-(*E*)-butenoyl-23-*epi*-26-deoxyactein (**3**), and 2′,12*β*-*O*-diacetylcimiracemonol-3-*O*-*β*-d-xylopyranoside (**4**), together with four known constituents (**5**–**8**) were isolated from the roots of *Cimicifuga foetida*. The new structures were elucidated by extensive spectroscopic analysis. In addition, compounds **7** and **8** showed significant Wnt signaling pathway inhibitory activity, with IC_50_ values of 3.33 and 13.34 μM, respectively, using the luciferase reporter gene assay.

## Introduction

Wnt signaling pathway plays an important role in numerous biological processes, including axis formation, cell fate specification, cell polarity determination, and cell migration [1]. Pathologically, Wnt signaling pathway is frequently aberrant in wide spectrum of malignancies, such as colon cancer, liver cancer, leukemia, melanoma, pancreatic cancer, and breast cancer [2]. Thus, screening inhibitors of Wnt signaling pathway has been considered as effective therapeutic strategies to combat cancer [3].

**Fig. 1:**
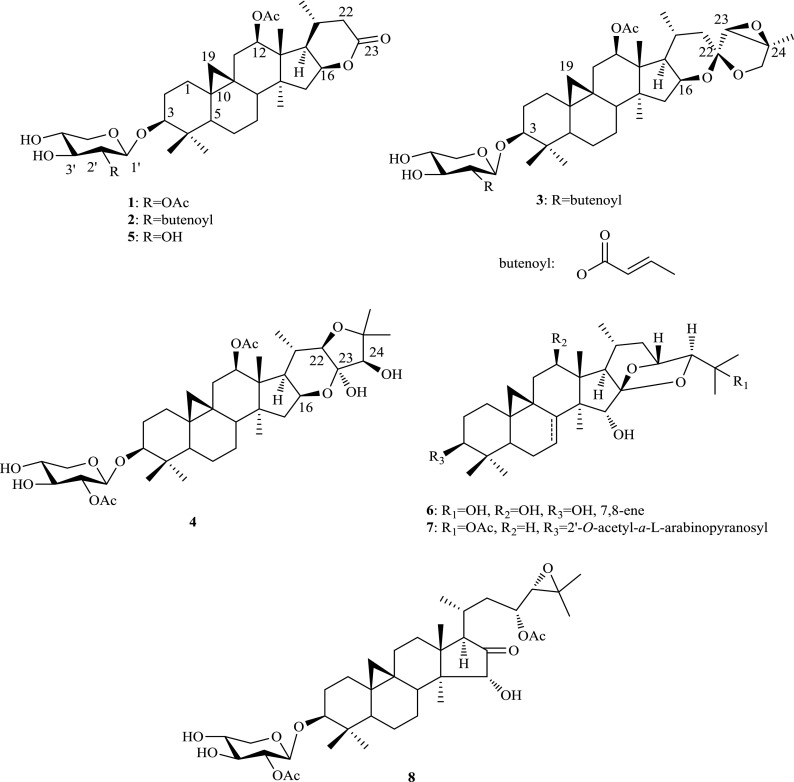
Structures of compounds **1**–**8**

**Fig. 2:**
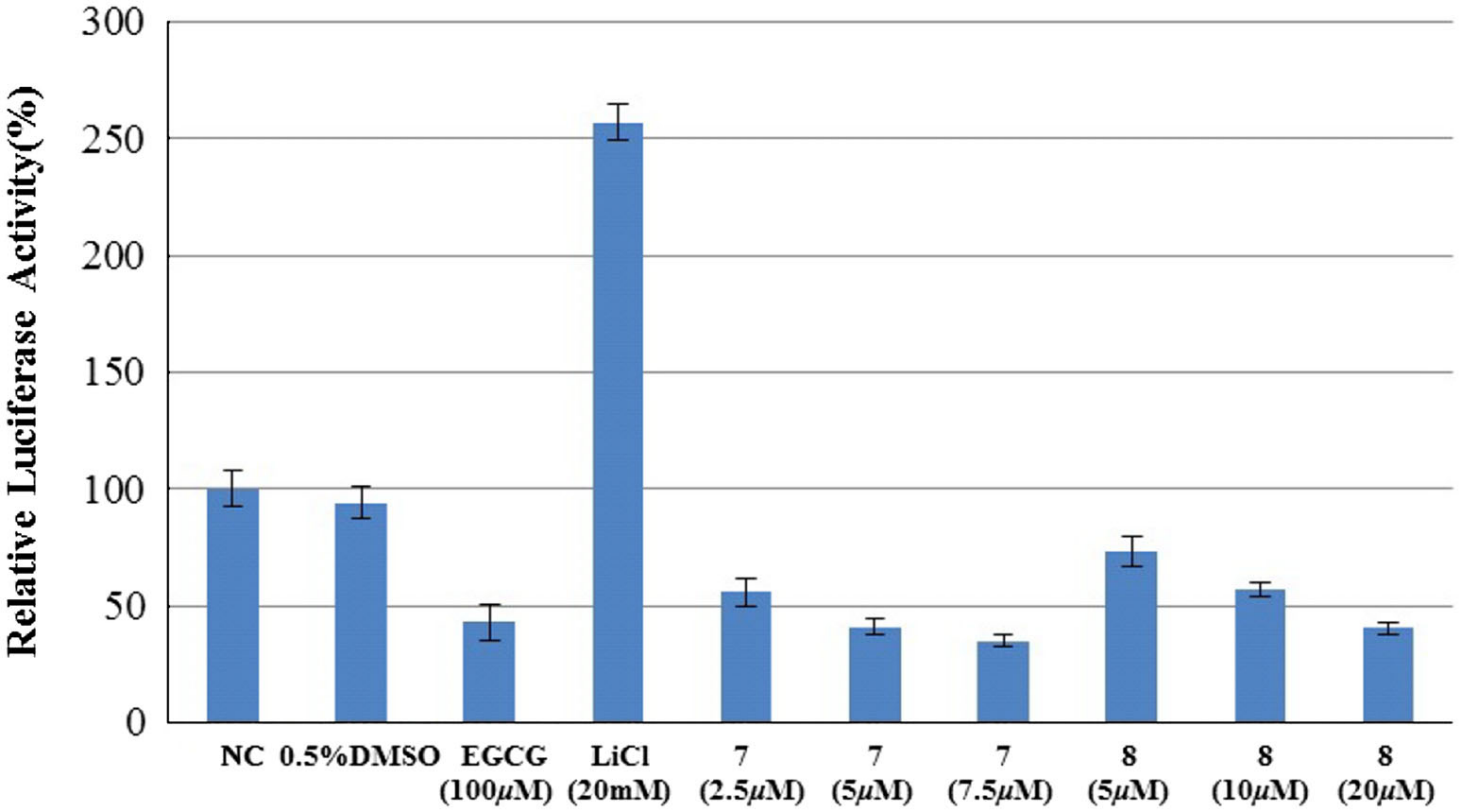
Inhibition of luciferase activity

Traditionally, the roots of *Cimicifuga foetida* have been used as a cooling and detoxifying remedy and officially listed in the Chinese Pharmacopoeia [4]. The theory of traditional Chinese medicine defines a tumor as a type of toxin. Base on this theory, we hypothesized that chemical constituents of *Cimicifuga* may have the effects against cancer, which led us to uncover a series of 9,19-cycloartane triterpenes. Many of them showed cytotoxic activity against tumor cell lines, in which five compounds isolated from *C. yunnanensis* induced apoptosis of MCF-7 cells via p53-dependent mitochondrial pathway, recently [5–14]. However, there have been no reports about the Wnt signaling pathway inhibitory activity of 9,19-cycloartane triterpenes by far. Therefore, to further screen inhibitors against Wnt signaling pathway from *Cimicifuga* spp., we carried out a study on the roots of *Cimicifuga foetid*a from Yulong County of Yunnan province. Consequently, four new compounds, cimilactone E (**1**), cimicilactone F (**2**), 2(*E*)-*O*-butenoyl-23-*epi*-26-deoxyactein (**3**), 2′-*O*-acetylcimirace-moside H (**4**), and four known compounds cimicilactone A (**5**) [15], 12*β*-hydroxy-7(8)-en-cimigenol (**6**) [16], cimicifoetiside B (**7**) [17], and 2′-*O*-acetyl cimiracemoside M (**8**) [18] (see Fig. 1) were isolated from *C. foetida*. Furthermore, all compounds isolated were evaluated for their inhibition of Wnt signaling pathway. Among them, compounds **7** and **8** showed significant inhibitory activities (see Fig. 2), with IC_50_ values of 3.33 and 13.34 μM, respectively. The present paper described the isolation, structure elucidation, and biological activities of aforementioned compounds.

## Results and Discussion

**Fig. 3:**
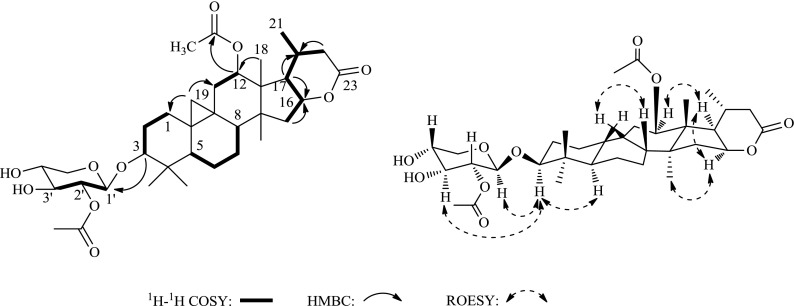
Key ^1^H-^1^H COSY, HMBC and ROESY correlations of compound **1**

Compound **1** was obtained as a white powder. The IR spectrum showed absorptions for hydroxyl groups at 3438 cm^−1^ and carbonyl groups at 1735 cm^−1^, respectively. Its molecular formula (C_35_H_52_O_10_) with ten degrees of unsaturation was deduced from HREIMS (*m/z* 632.3533 [M]^+^; calcd. 632.3560). In ^1^H NMR spectrum (Table 1), the characteristic cyclopropane methylene signals at *δ*_H_ 0.15 and 0.50 (each 1H, d, *J* = 4.3 Hz), one secondary methyl signal at *δ*_H_ 0.98 (d, *J* = 6.4 Hz), four tertiary methyl groups at *δ*_H_ 0.84–1.26 (each 3H, s), an anomeric proton at *δ*_H_ 4.83 (1H, d, *J* = 8.0 Hz) and two acetyl methyl groups at *δ*_H_ 2.16 and 2.18 (each 3H, s) were observed. The ^13^C NMR and DEPT spectra of **1** exhibited 35 signals, of which 26 were attributed to the aglycon, five to a pentose residue, and four to two acetyl groups. The ^13^C NMR and DEPT spectrum of the aglycon of **1** also showed an ester carbonyl carbon at *δ*_C_ 174.2 and two acetoxy carbonyl groups at *δ*_C_ 171.1 and 170.5. The aforementioned data suggested that **1** was a 9,19-cycloartane tetranortriterpene glycoside with three carbonyl groups. The NMR spectroscopic data of **1** (Table 1) closely resembled that of cimilactone A (**5**), except for the presence of an additional acetoxy group. In the HMBC spectrum (Fig. 3), a correlation was observed between the proton at *δ*_H_ 4.87 (1H, d, *J* = 8.0 Hz, H-1′) and the methine carbon at *δ*_C_ 88.8 (C-3), suggesting that the sugar moiety was located at C-3. In ^1^H-^1^H COSY spectrum, the correlations of a downfield resonance at *δ*_H_ 5.58 (1H, dd, *J* = 8.0 and 9.0 Hz, H-2′) with H-3′ (*δ*_H_ 4.20, m) and H-1′ (*δ*_H_ 4.83, d, *J* = 8.0 Hz), together with the HMBC correlations from the proton resonance (H-2′) to a carbonyl group (*δ*_C_ 170.5, s) located the acetoxy group at C-2′. The sugar obtained after acid hydrolysis was identified as d-xylose by comparing its TLC and specific rotation with a standard. The relative configurations of H-3, H-5, H-12, H-16, and H-17 were established as in the *α*-orientation on the basis of the ROESY correlations of H-1′/H-3; H-3/H-5; H-12/H-17; H-16/H-17 and H-17/Me-18 (Fig. 3). Therefore, the structure of **1** was determined to be (3*β*,12*β*,16*β*)-12,2′–diacetoxy-3-hydroxy-24,25,26,27-tetranor-cycloartan-23,16-olide 3-*O*-*β*-d-xylopyranside (**1**), named cimilactone E.

**Table 1:** NMR data of compounds **1** and **2** (*δ* in ppm and *J* in Hz)

Position	**1** ^a^		**2** ^a^		**3** ^a^		**4** ^a^	
	***δ***_C_	***δ***_H_	***δ***_C_	***δ***_H_	***δ***_C_	***δ***_H_	***δ***_C_	***δ***_H_
1	32.4 t	1.06 m 1.45 m	32.2 t	1.06 m 1.46 m	32.2 t	1.07 m 1.46 m	32.2 t	1.06 m 1.44 m
2	30.2 t	1.80 m 2.19 m	30.2 t	1.82 m 2.21 m	30.2 t	1.80 m 2.20 m	30.2 t	1.78 m 2.19 m
3	88.6 d	3.34 dd (4.4, 11.5)	88.7 d	3.37 dd (4.2, 11.4)	88.7 d	3.34 dd (4.2, 11.6)	88.7 d	3.34 dd (4.2, 11.4)
4	41.3 s		41.4 s		41.3 s		41.3 s	
5	47.2 d	1.22 dd (4.3, 11.2)	47.2 d	1.22 m	47.2 d	1.18 m	47.3 d	1.22 m
6	20.8 s	0.69 m 1.47 m	20.8 s	0.67 m 1.46 m	20.4 t	0.56 m 1.37 m	20.4 t	0.68 m 1.44 m
7	26.1 t	0.91 m 1.22 m	26.1 t	0.90 m 1.22 m	26.1 t	0.87 m 1.16 m	26.2 t	0.90 m 1.24 m
8	46.5 d	1.57 dd (5.0, 12.1)	46.5 d	1.55 dd (4.8, 12.0)	46.2 d	1.53 dd (5.4, 12.0)	46.2 d	1.54 dd (5.4, 9.4)
9	20.5 s		20.5 s		20.7 s		20.9 s	
10	27.2 s		27.2 s		26.9 s		26.9 s	
11	36.8 t	1.14 dd (3.6, 16.2) 2.71 dd (8.9, 16.2)	36.8 t	1.13 m 2.71 dd (9.0, 16.2)	37.1 t	1.16 m 2.71 m	37.2 t	1.14 m 2.73 dd(9.0, 16.2)
12	77.0 d	5.07 dd (3.6, 8.9)	77.0 d	5.06 dd (3.6, 9.0)	77.4 d	5.09 m	77.4 d	5.15 m
13	48.6 s		48.6 s		49.2 s		49.8 s	
14	49.0 s		49.0 s		48.1 s		48.5 s	
15	44.2 t	1.84 dd (5.6, 13.6) 2.01 dd (8.0, 13.6)	44.2 t	1.83 m 1.99bdd (8.4, 13.8)	44.6 t	1.76 m 1.88 dd (7.8, 12.6)	43.5 t	1.76 dd (8.4, 11.4) 1.94 dd (7.8, 12.0)
16	80.8 d	4.82 m	80.8 d	4.81 m	74.9 d	4.24 m	72.5 d	5.03 dd (7.8, 16.2)
17	54.1 d	2.15 m	54.0 d	2.15 m	56.6 d	1.77 m	52.9 d	1.82 dd (10.2, 19.2)
18	13.7 q	1.26 s	13.7 q	1.24 s	14.7 q	1.47 s	14.2 q	1.37 s
19	30.1	0.15 d (4.3) 0.50 d (4.3)	30.1 t	0.15 d (4.2) 0.48 d (4.2)	29.9 t	0.14 d (4.2) 0.44 d (4.2)	30.1 t	0.15 d (3.6) 0.46 d (3.6)
20	27.1 d	2.01 m	27.1 d	2.05 m	23.7 q	2.24 m	34.9 d	2.29 m
21	22.3 q	0.98 d (6.4)	22.3 q	0.97 d (6.0)	21.7 q	1.02 d (6.6)	18.9 q	1.35 d (6.0)
22	39.1 t	2.28 m 2.49 dd (3.5, 14.6)	39.0 t	2.28 m 2.48 dd (3.6, 15.0)	37.9 t	1.44 m 1.59 dd (3.0, 13.8)	87.1 d	3.91 d (10.8)
23	174.2 s		174.2 s		106.3 s		106.0 s	
24					62.6 d	3.68 s	83.7 d	4.24 s
25					62.9 s		83.7 s	
26					68.5 t	3.63 d (10.2) 4.06 d (10.2)	28.2 q	1.79 s
27					13.9 t	1.41 s	25.3 q	1.72 s
28	20.0 q	0.84 s	19.9 q	0.83 s	20.1 q	0.83 s	20.1 q	0.85 s
29	25.8 q	1.10 s	25.8 q	1.12 s	25.8 q	1.10 s	25.8 q	1.10 s
30	15.5 q	0.94 s	15.5 q	0.94 s	15.5 q	0.91 s	15.5 q	0.92 s
3-Xyl								
1′	105.0 d	4.83 d (8.0)	105.2 d	4.89 d (7.8)	105.2 d	4.88 d (7.8)	105.0 d	4.82 d (7.8)
2′	76.1 d	5.58 dd (8.0, 9.0)	75.8 d	5.68 dd (7.9, 9.0)	75.8 d	5.67 dd (7.8, 10.2)	76.1 d	5.58 t (9.0)
3′	76.6 d	4.20 m	76.8 d	4.26 m	76.8 d	4.23 m	76.6 d	4.19 m
4′	71.7 d	4.23 m	71.8 d	4.26 m	71.7 d	4.25 m	71.7 d	4.22 m
5′	67.5 t	3.71 dd (9.9, 10.8) 4.33 dd (5.0, 10.8)	67.6 t	3.74 m 4.35 dd (4.2, 11.4)	67.6 t	3.74 t (10.8) 4.35 dd (4.2, 10.8)	67.5 t	3.70 t (10.8) 4.32 dd (4.2, 10.8)
12-COCH_3_	171.1 s		171.1 s		171.1 s		171.1 s	
12-COCH_3_	21.9 q	2.18 s	21.9 q	2.16 s	22.1 q	2.15 s	22.1 q	2.10 s
2′-COCH_3_	170.5 s						170.5 s	
2′-COCH_3_	21.7 q	2.16 s					21.7 q	2.17 s
2′-butenoyl								
1″			166.2 s		166.2 s			
2″			123.6 d	6.12 d (15.6)	123.6 d	6.11 dd (1.6, 15.5)		
3″			145.4 d	7.14 m	145.4 d	7.12 m		
4″			18.2 q	1.68 d (6.6)	18.2 q	16.7 d (6.6)		

^a^Measured in pyridine-*d*_5_

Compound **2** was obtained as a white powder. The IR spectrum showed absorptions for hydroxyl (3442 cm^−1^), carbonyl group (1732 cm^−1^), and double bond (1632 cm^−1^), respectively. The HREIMS spectrum gave an [M]^+^ ion peak at *m/z* 658.3713 (calcd 658.3717), consistent with the molecular formula C_37_H_54_O_10_, requiring eleven rings or sites of unsaturation. The NMR data of **2** was similar to cimilactone E (**1**), except for the resonances of the sugar moiety. The ^13^C NMR spectrum revealed carbons assignable to a 2-butenoyl moiety at *δ*_C_ 165.8 (s), 123.6 (d), 144.8 (d). Besides, the correlation from the proton resonance (*δ*_H_ 5.68, dd, *J* = 7.9 and 9.0 Hz, H-2′) to the carbonyl group (*δ*_C_ 166.2, s, C-1″) in the HMBC spectrum located the 2-butenoyl group at C-2′, which was further confirmed by the ^1^H-^1^H COSY correlations of the proton at *δ*_H_ 5.68 (H-2′) with H-1′ and H-3′. In addition, the coupling constant (*J* = 15.6 Hz) of the two olefinic protons at *δ*_H_ 6.12 (H-2″) and *δ*_H_ 7.14 (H-3″) confirmed the *E*-geometry of a double bond in the 2-butenoyl moiety. The sugar obtained after acid hydrolysis was confirmed as d-xylose by comparing its TLC and specific rotation with a standard. The relative configurations of H-3, H-5, H-12, H-16, and H-17 were proposed as *α*-orientation, respectively, by the same way as that of **1**. Therefore, the structure of **2** was identified as (3*β*,12*β*,16*β*)-12-acetoxy-3-hydroxy-24,25,26,27-tetranor-cycloartan-23,16-olide-3-*O*-{2′-*O*-(*E*)-2″-butenoyl}-*β*-d-xylopyran-side **(2)**, named cimilactone F.

Compound **3**, was obtained as white powder, which gave an [M]^+^ ion at *m/z* 728.4120 [M]^+^ (calcd 728.4120) in HREIMS, consistent with the molecular formula of C_41_H_60_O_11_. The IR spectra showed absorption bands for hydroxyl groups at 3442 cm^−1^, carbonyl groups at 1730 cm^−1^ and double bond at 1632 cm^−1^, respectively. Typical proton signals of a cyclopropane methylene group (*δ*_H_ 0.14 and 0.44, each 1H, d, *J* = 4.3 Hz); five tertiary methyl groups (*δ*_H_ 0.83–1.47, each 3H, s); two secondary methyl group [(*δ*_H_ 1.02 (3H, d, *J* = 6.0 Hz) and 1.67 (3H, d, *J* = 6.6 Hz)]; one acetyl methyl group (*δ*_H_ 2.15, 3H, s); one 2(*E*)-butenoyl group [*δ*_H_ 6.11 (1H, dd, *J* = 1.6 and 15.5 Hz), 7.12 (m) and 16.7 (3H, d, *J* = 6.6 Hz)], and an anomeric proton (*δ*_H_ 4.88, 1H, d, *J* = 7.8 Hz) were observed in the ^1^H NMR spectrum (Table 1). The ^13^C NMR and DEPT spectrum (Table 1) of **3** exhibited 41 signals, of which 30 were attributed to the aglycon, five to a pentose residue, two to an acetyl group, and four to an 2-butenoyl group. Additionally, the aglycon of **3** also showed a hemiketal moiety at *δ*_C_ 74.9 (d), 106.3 (s) and 68.5 (t), and an epoxyethane signals at *δ*_C_ 62.6 (d) and 62.9 (s), which indicated that the aglycon of **3** was similar with 23-*epi*-deoxyacteol. A comparison of the spectroscopic data of **3** with those of 23-*epi*-26-deoxyactein showed that **3** closely resembles of it except for the presence of another tetra-carbon unit (2-butenoyl group) [19]. Additionally, the *E*-geometry of a double bond in the 2-butenoyl was confirmed in the same way with that of **2**. In ^1^H-^1^H COSY spectrum, the correlations of a resonance at *δ*_H_ 7.12 (m, H-3″) with H-2″ (*δ*_H_ 6.11, dd, *J* = 1.6 and 15.6 Hz) and H-4″ (*δ*_H_ 1.67, d, *J* = 6.6 Hz), together with the HMBC correlation from the proton resonance (*δ*_H_ 5.67, dd, *J* = 7.8 and 10.2 Hz, H-2′) to a carbonyl group (*δ*_C_ 166.2, s) located the 2(*E*)-butenoyl group at C-2′. Thus, **3** was characterized as 2′-*O*-2(*E*)-butenoyl-23-*epi*-26-deoxyactein (**3**).

Compound **4** was obtained as a white powder. The IR spectrum showed absorptions for hydroxyl (3439 cm^−1^), carbonyl groups (1730 cm^−1^), respectively. Its molecular formula (C_39_H_60_O_12_) with ten degrees of unsaturation was deduced from the analyses of ^13^C NMR and HREIMS data (*m/z* 720.4078 [M]^+^; calcd. 720.4085). In ^1^H NMR spectrum (Table 1), the signals due to a cyclopropane methylene group (*δ*_H_ 0.15 and 0.46, each 1H, d, *J* = 3.6 Hz); six tertiary methyl groups (*δ*_H_ 0.85–1.79, each 3H, s); one secondary methyl group (*δ*_H_ 1.35, 3H, d, *J* = 6.0 Hz); two acetyl methyl group (*δ*_H_ 2.17 and 2.10, each 3H, s); and an anomeric proton (*δ*_H_ 4.82, 1H, d, *J* = 7.8 Hz) were observed. The ^13^C NMR and DEPT spectrum (Table 1) of **4** exhibited 39 signals, of which 30 were attributed to the aglycon, five to a pentose residue, and four to two acetyl groups. All above showed **4** was similar to cimiracemoside H, except for the presence of an additional acetoxy group. In ^1^H-^1^H COSY spectrum, the correlations of a resonance at *δ*_H_ 4.19 (m, H-3′) with H-2′ (*δ*_H_ 5.58, t, *J* = 9.0 Hz) and H-4′ (*δ*_H_ 4.22, m), together with the HMBC correlation from a carbonyl group (*δ*_C_ 170.5, 3H, s) to the proton resonance (*δ*_H_ 5.58, H-2′) located the acetoxy group at C-2′. The ROESY correlations of H-17/H-22 and Me-21/H-24 proved H-22 in the *α*-orientation and H-24 as *R* configuration. Therefore, **4** was characterized as (3*β*,12*β*,16*β*,20*S*,22*R*,23*S*,24*R*)-16:23; 22:25-diepoxy-12,2′-diacetoxy-3,23,24-trihydroxy-9, 19-cyclocanostane-3-*O*-*β*-d-xylopyranoside (**4**), named 2′-*O*-acetyl cimiracemoside H.

As noted in the introduction, the roots of *C. foetida* have been used as cooling and detoxification agents by Chinese people since ancient time. Previous reports have shown that many pure 9,19 cycloartane triterpenoids isolated from this species exhibited cytotoxic activity against 11 tumor cell lines (including HepG2, MDA-MB-A231, HL-60, SMMC-7721, A549, SK-BR-3, PANC-1, K562, U933, HEG-2, and SGC-7091) in vitro, respectively [5–7, 17, 20, 21]. But there is no report on inhibition of Wnt signaling pathway. The pure components isolated in the present paper were screened against Wnt signaling pathway using the luciferase reporter gene assay. The known compounds **7** and **8** showed notable activity with the IC_50_ values of 3.33 and 13.34 μM, respectively. To the best of our knowledge, this is the first time to report the inhibitory activity against Wnt signaling pathway of 9,19-cycloartane triterpenes. These data suggested that some chemical constituents from *C. foetida* might be valuable to against tumorigenesis through inhibition to Wnt signaling pathway.

## Experiments Section

### General Experimental Procedures

UV spectra were recorded in MeOH on a shimadizu UV-210A spectrometer. IR spectra were recorded on Shimadzu IR-450 spectrometer with KBr disc. Optical rotations were measured a Horiba SEAP-300 polarimeter. ^1^H NMR and ^13^C NMR spectra were recorded using a Bruker AM-600 spectrometer with TMS as internal standard, operating at 600 and 150 MHz, respectively. All compounds were measured in solvents pyridine-*d*_*5*_. ESIMS and HRESIMS were carried out on a Waters Autospec Premier-P776 spectrometer. TLC analysis was performed on silica gel GF_254_ plate (Qingdao Marine Chemical, Inc.). Lichroprep RP-18 (40–63 μm, Merck) and silica gel (200–300 mesh) was used for column chromatography. Semipreparative HPLC was carried out on an Agilent 1260 liquid chromatograph with a ZORBAX SB C-18 column (9.4 × 250 mm,5 μm,) and a ZORBAX XDB C-18 column (9.4 × 250 mm, 5 μm).

### Plant Materials

The roots of *C. foetida* (82 kg) were collected from Yulong County of Yunnan province of China in September 2010 and authenticated by Prof. Shen-Ji Pei of Kunming Institute of Botany, where a voucher specimen (KUN No. 20100906) is deposited.

### Extraction and Isolation

The air-dried roots of *C. foetida* (82 kg) were crushed with a blender and refluxed with 95 % MeOH for three times (5 h, each). The residue yield by removal of the solvent was dissolved in water to form a suspension. The aqueous suspension was successively partitioned with EtOAc and n-BuOH. The EtOAc (5.6 kg) fraction was absorbed on 12 kg silica gel and chromatographed on a prepacked (120 kg) silica gel column, eluting stepwise with CHCl_3_-MeOH (CHCl_3_, 100:1, 50:1, 20:1, 5:1) to give five fractions (I–V). Fr.IV (350 g) was subjected to silica gel chromatograph eluted with CHCl_3_-acetone (10: 1) to give five sub-fractions (Fr.IV. 1–5). Fr.IV.4 (0.5 g) was chromatographed repeatedly over HPLC (SB C-18 column, CH_3_CN-H_2_O, 7:3) to obtain compound **7** (5 mg) successively. Fr.IV.5 (5 g) was applied repeatedly to CC over RP-18 gel (60, 70, 80 and 90 % MeOH-H_2_O) to give fractions IV.5.1–4. Fr.IV.5.2 (60 mg) afforded compound **6** (5 mg), after repeated elution with a CHCl_3_-acetone (5:1, 3:2) system over silica gel CC and a CH_3_CN-H_2_O (65 %) over HPLC (SB C-18 column). Fr.IV.5.3 (450 mg) was separated by column chromatograph eluted with CHCl_3_-acetone (3:1), and by HPLC (SB C-18 column) with CH_3_CN/H_2_O (45 %) to obtain compounds **1** (10 mg), **4** (2.2 mg) and **5** (3 mg), respectively. Fr.IV.5. 4 (380 mg) was also separated by column chromatograph eluted with CHCl_3_-acetone (3:1), then HPLC (XDB C-18 column) with CH_3_CN-H_2_O (65 %) to yield compounds **2** (1.6 mg), **3** (1.8 mg) and **8** (5 mg).

Compound **1**: white powder (MeOH); $[\alpha ]_{\rm{D}}^{20}$: −41.67 (*c* 0.24, MeOH); UV (MeOH) *λ*_max_ (log ε): 209 (2.32) nm; IR (KBr) *v*_max_: 3438 (OH), 2964, 1735 (C=O), 1631(C=C), 1442, 1375, 1242, 1032, 982 cm^−1^; positive HRESIMS *m/z* 632.3533 [M]^+^, (C_35_H_52_O_10_, calcd. 632.3560), ^1^H and ^13^C NMR data, see Table 1.

Compound **2**: white powder (MeOH); $[\alpha ]_{\rm{D}}^{20}$: −90.67 (*c* 0.06, MeOH); UV (MeOH) *λ*_max_ (log ε): 206 (4.29) nm; IR (KBr) *v*_max_: 3442 (OH), 2963, 2936, 1732 (C=O), 1655(C=C), 1444, 1365, 1245, 1184, 1072, 984 cm^−1^; positive HRESIMS *m/z* 658.3713 [M]^+^, (C_37_H_54_O_10_, calcd. 658.3717), ^1^H and ^13^C NMR data, see Table 1.

Compound **3**: white powder (MeOH); $[\alpha ]_{\rm D}^{20}$: −49.18 (*c* 0.18, MeOH); UV (MeOH) *λ*_max_ (log ε): 206 (4.14) nm; IR (KBr) *v*_max_: 3441 (OH), 2932, 1730 (C=O), 1651(C=C), 1443, 1365, 1250, 1184, 1072, 1032, 982 cm^−1^; positive HRESIMS *m/z* 728.4120 [M]^+^, (C_41_H_60_O_11_, calcd. 728.4136), ^1^H and ^13^C NMR data, see Table 1.

Compound **4**: white powder (MeOH); $[\alpha ]_{D}^{20}$: −19.67 (*c* 0.13, MeOH); UV (MeOH) *λ*_max_ (log ε): 201 (3.58) nm; IR (KBr) *v*_max_: 3439 (OH), 2939, 1735 (C=O), 1632(C=C), 1462, 1377, 1244, 1159, 1047, 981, 602–576 cm^−1^; positive HREIMS *m/z* 720.4078 [M]^+^, (C_39_H_60_O_12_, calcd. 720.4085), ^1^H and ^13^C NMR data, see Table 1.

The known compounds, cimilactone A (**5**), 12*β*-hydroxy-7(8)-en-cimigenol (**6**), cmicifoetiside B **(7)** and 2′-*O*-acetyl cimiracemoside M (**8**) were identified by comparing their physical and spectroscopic data with reported data.

### Acidic Hydrolysis of **1**–**4**

A solution of each new compound (1 mg) in 0.5 N HCl (3 ml) was refluxed for 4 h. The reaction mixture was diluted in 10 mL water and extracted with chloroform. After separating the organic layer, the aqueous phase was neutralized with Ag_2_CO_3_ to obtain some white precipitate. The precipitate residue was dissolved in pyridine and analyzed by TLC in n-BuOH-acetone-H2O (4:3:1, v/v), which had the same *R*_*f*_ value with D-(+)-xylose [22].

### Luciferase Activity

The Wnt signaling inhibitory activity of the eight 9, 19-cycloartane triterpenes **(1**–**8)** using the luciferase reporter gene assay as previously described [2]. Briefly, HEK293W cells were seeded in 96 well plate, and the luciferase activities were measured after incubation with the triterpenes for 24 h, using the Dual-Lucy Assay Kit (Promega) according to the manufacturer’s instructions.
